# A review of NTRK fusions in cancer

**DOI:** 10.1016/j.amsu.2022.103893

**Published:** 2022-06-13

**Authors:** Cristina Alexandra Manea, Dumitru Cristinel Badiu, Ioan Cristian Ploscaru, Anca Zgura, Xenia Bacinschi, Catalin Gabriel Smarandache, Dragos Serban, Cristian Gabriel Popescu, Valentin Titus Grigorean, Vladimir Botnarciuc

**Affiliations:** a Military Emergency Hospital Constanta, Ovidius University of Medicine and Pharmacy, Constanta, Romania; b General Surgery Department, Bagdasar Arseni Clinical Emergency Hospital, “Carol Davila” University of Medicine and Pharmacy, Bucharest, Romania; c Ovidius University of Medicine and Pharmacy, Constanta, Romania; d Department of Oncology-Radiotherapy, Prof. Dr. Alexandru Trestioreanu Institute of Oncology, Carol Davila University of Medicine and Pharmacy, Bucharest, Romania; e Department of General Surgery, Emergency University Bucharest, “Carol Davila” University of Medicine and Pharmacy, Bucharest, Romania

**Keywords:** NTRK gene, Carcinogenesis, Cancer, Review

## Abstract

The family of the neurotrophic tropomyosin kinase receptors (NTRK or TRK) is a part of the transmembrane tyrosine kinases responsible for neuronal development. The members of this receptor family are TRKA, TRKB and TRKC and they are encoded by the genes *NTRK1*, *NTRK2* and *NTRK3*. Alterations of *NTRK* genes can induce carcinogenesis both in neurogenic and non-neurogenic cells. The prevalence of *NTRK* gene fusion is under 1% in solid tumors, but is highly encountered in rare tumors. The presence of *NTRK 1* gene fusion is associated, in some types of neoplasia, with a favorable evolution, but the presence of NTRK 2 may be associated with a poor prognosis. The identification of cancer patients harboring *NTRK* gene fusions is constantly growing, especially with the advent of NTRK inhibitors. This has promisingly provided a rationale for personalized therapeutics that improved outcomes in settings with this signature.

## Highlights

• NTRK is a family of receptors involved in neural development.
• The role of NTRK fusions in cancer was recently uncovered.
• The list of solid cancers harboring NTRK fusions is expanding.
• NTRK gene fusions were recently emerged as potential predictive biomarkers of therapy resistance and also of survival.
• NTRK gene fusions are being targeted for cancer drug discovery.

## 1 Introduction

The family of the neurotrophic tropomyosin kinase receptors (NTRK or TRK) are a part of the transmembrane tyrosine kinases responsible for neuronal development. The three members of this receptor family TRKA, TRKB and TRKC, encoded by the genes *NTRK1*, *NTRK2* and *NTRK3*, have an extracellular binding domain, a transmembrane region and an intracellular kinase domain [1]. The ligands of NTRK receptors are called neurotrophins and have a particular site for binding. The nerve growth factor (NGF) binds with NTRK1, the brain-derived neurotrophic factor (BDNF) together with neurotrophin 4 and 5 bind with NTRK2 and neurotrophin 3 binds with NTRK3 but also with NTRK1. The binding of the neutrophins with their receptors determines the activation of the effectors within NTRK signaling such as C- γ phospholipase, mitogen activated protein kinase or phosphatidylinositol 3 kinase. These receptors are protemers for the proliferation and survival of the neural cells [2]. Alterations of *NTRK* genes can induce carcinogenesis both in neurogenic and non-neurogenic cells. These alterations are being used as predictive biomarkers for emerging targeted agents but as genetic alterations; they are very rarely present in solid tumors. A study by Li et al. [3] that enrolled 173 patients with pulmonary cancer revealed NTRK alterations in 8 patients including 4 N*T*RK gene mutations, 2 NTRK gene fusions, and 2 NTRK gene deletions. The prevalence of *NTRK* gene fusion is under 1% in solid tumors, but is highly seen in rare tumors such as mammary secretory carcinoma, mammary analogue secretory carcinoma of the salivary gland, congenital infantile fibrosarcoma (fusion prevalence is almost 100%), and other rarer tumors. *NTRK* fusion is present in approximately 40% of high-grade non-brainstem pediatric gliomas. These fusions are more likely present in *NTRK1* and *NTRK2* genes [2,4].

## 2 Epidemiology of *NTRK* gene fusion in cancer

*NTRK* gene fusion is present in several cancers including both adult and pediatric populations. Two categories of *NTRK* fusion cancers can be individualized, rare cancers where the frequency of *NTRK* gene fusion is approximately 80% and common cancers having5 to 25% alterations of this gene (Table 1).

**Table 1:** **NTRK gene fusions in cancer types**.

NTRK gene fusion	Type of cancer	References
NTRK1		
LMNA-NTRK1	Colorectal cancer	[5]
	Sarcoma	[6]
	Spitzoid melanoma	[7]
	Adolescent and young adult sarcoma	[8]
	Infantile congenital fibrosarcoma	[9]
TPM3 – NTRK1	Colorectal	[10]
	Papillary thyroid carcinoma	[11]
	Glioblastoma	[12]
SQSTM1-NTRK1	Non-small cell lung carcinoma	[13,14]
NFASC-NTRK1	Multiform glioblastoma	[15,16]
BCAN-NTRK1	Multiform glioblastoma	[17,18]
PPL-NTRK1	Thyroid carcinoma	[19]
RFWD2-NTRK1	Large cell neuroendocrine lung cancer	[20]
CD74-NTRK1	Lung adenocarcinoma	[21]
MPRIP-NTRK1	Lung adenocarcinoma	[22]
RABGAP1L-NTRK1	Invasive cervical cancer	[23]
TFG-NTRK1	Thyroid carcinoma	[24]
TP53-NTRK1	Spitzoid melanoma	[25]
**NTRK2**		
Unknown– NTRK2	Appendix adenocarcinoma	[26]
AFAP1- NTRK2	Low-grade glioma	[27]
AGBL4-NTRK2	Glioblastoma	[28]
NACC2-NTRK2	Pilocytic astrocytoma	[29]
PAN3-NTRK2	Head and neck squamous carcinoma	[28]
QKI – NTRK2	Pilocytic astrocytoma	[29]
TRIM 24 – NTRK2	Lung adenocarcinoma	[28]
VCL – NTRK2	Glioblastoma	[28]
**NTRK3**		
ETV6 – NTRK3	Glioblastoma, ductal carcinoma, fibrosarcoma, congenital mesoblastic nephroma, irradiation associated-thyroid cancer, papillary thyroid cancer, GIST, colorectal cancer, secretory mammary pulmonary analogue cancer, acute myeloid leukemia	[[30], [31], [32], [33], [34], [35], [36]]

Notably, *NTRK* gene fusion is present in 90–100% of mammary analogue secretory carcinoma of the breast, in >90% of mammary secretory carcinoma, in 91–100% of fibrosarcoma and in 83% of congenital mesoblastic nephroma. Although the frequency of *NTRK* fusion is smaller; it is present in 14.5% of thyroid irradiation associated-cancer, 26% of papillary thyroid cancer and 16% of spitzoid tumors. In common cancers, the frequency of *NTRK* gene fusion is less than 5%. Head and neck neoplasms have approximately 0.2% of this alteration, 0.2–0.3% were found in pulmonary cancer, 0.7–1.5% in colorectal cancer, 0.3% in cutaneous melanoma, and 1% in sarcoma [6].

## 3 Methods for NTRK fusion detection

Immunohistochemistry to examine NTRK protein expression has a number of advantages. It is accessible and affordable, easy to implement, and is already routinely used in many diagnostic laboratories. It also has a lower cost than other methods, requires a single-colored blade and has a fast turnover. The most used clone is EPR17341 (Abcam and Roche/Ventana) which reacts with a peptide in the C-terminal portion of TRK-A, TRK-B and TRK-C, thus causing a reaction with any fusion of *NTRK* genes. Positive staining was established to be present in at least 1% of tumor cells. The sensitivity for *NTRK 1* and *NTRK 2* fusions is estimated at approximately 96% and 100%, respectively, while for *NTRK3* around 79% [1].

Fluorescence *in situ* hybridization (FISH method) is often used in laboratories to detect oncogene fusion in solid tumors. This method can detect large structural variations in DNA. There is currently a diagnostic test available for the ETV6 gene which is most commonly encountered as a fusion oncogene with *NTRK3*. In positive cases, a green signal may be seen when the structure is affected at the 3 ′centromeric end and an orange signal when structural change occurs at the end 5'. Other FISH kits are being developed and will be available in the future for different *NTRK* gene fusion partners [1].

Reverse transcriptase chain polymerase reaction (RT–PCR) can be used to detect the presence of transcribed RNA, thus able to evaluate and detect qualitatively and quantitatively the presence of a single fusion oncogene for which both fusion partners are known [1]. In the case of *NTRK* gene fusion, due to multiple fusion partners, the utility of RT-PCR is limited and it is used mainly for *ETV6-NTRK3* fusion detection [1].

The next-generation DNA sequencing (NGS) technology can also be used and it is based on the extraction of tumor DNA from tissue fixed in formalin-paraffin and subsequently sequenced to determine if there are specific genetic alterations in tumor tissues. There are also disadvantages of this method, such as the length of the gene introns. *NTRK3* gene introns have a length of about 193 KB and are too long for an accurate sequencing and also that fact of the presence of new partner genes, not yet identified yet, of fusions with *NTRK* genes [1].

On the other hand, transcriptomics using next-generation RNA sequencing is advantageous over NGS of DNA. Introns are sequenced outside the RNA, which eliminates the technical limitation given by “covering” the introns. In addition, by this method, the detection of RNA fusion ensures the direct evidence that these are functionally transcribed. Transcribed fusions can be detected more accurately in the RNA of tumors with small pieces of purity, due to the fact that gene fusions are of greater expression in the tumor tissues [1].

## 4 Oncological aspects of *NTRK* gene fusions

### 4.1 Lung cancer

Fusions of the *NTRK* gene are found to a small extent in lung cancer, with an incidence of around 0.1% [37]. Regarding the *NTRK1* and *NTRK2* genes, it was noticed that they are found mainly in the normal epidermis, oral mucosa and epidermal carcinomas and particularly *NTRK2* in the lung, the liver and the heart tissue [7].

*NTRK1* and *NTRK2* are preferentially expressed in squamous cell carcinoma as compared to other histological types, especially adenocarcinoma. Data from studies in patients with primary lung cancer showed that *NTRK1* has a high specificity (92.8%) in a group of 686 patients with squamous cell carcinoma, but has a low sensitivity 71.6%. In the same group of patients, *NTRK2* had a specificity of 96.4%, but also with a low sensitivity of 51.3%. Accumulating data from several studies demonstrated that *NTRK2* may be a useful biomarker for squamous cell proliferation. Moreover, a study performed on 60 patients with non-microcellular lung cancer, found that *NTRK2* expression was 66.7%and remarkably, a statistically significant correlation between *NTRK2* gene expression and TNM stage and secondary lymphatic determinations was also observed [7]. However, another study found that the presence of *NTRK1* does not significantly influence the prognosis of squamous cell carcinoma or lung adenocarcinoma, but is a positive prognostic factor for patients with squamous cell carcinoma regardless of gender, age, stage or smoking status [8]. The association between the presence of *NTRK2* and a good prognosis in patients with squamous cell carcinoma may suggest an important role of this gene in the biology of this type of neoplasia [8].

### 4.2 Colorectal and thyroid cancers

The first fusion of the *NTRK* gene was discovered in 1986 in a patient with colorectal cancer. Since then, this fusion has been reported in about 2% of patients with this type of cancer. Later, multiple studies have concluded that *TPM3-NTRK1* fusion is found mainly in colorectal carcinoma, in addition to the relatively high frequency of *TPR-NTRK1* fusion [37]. Among the characteristics of patients with colorectal cancer who also have fusion of the *NTRK* gene are female sex, location in the right colon and microsatellite instability phenotype (MSI) [9]. In thyroid cancer, two years after the identification of the fusion of the *NTRK* gene in colorectal cancer, the fusion was also identified in 4 patients with papillary thyroid carcinoma. Subsequent studies have shown that in this type of carcinoma, *NTRK* rearrangements take place in the tyrosine kinase domain of the *NTRK1* gene and the 5 ′end of the 3 partners (TRKT1, TRKT2 and TRKT3). Currently the estimated incidence of *NTRK1* gene fusion in papillary thyroid carcinoma is <12%. In particular, *ETV-NTRK3* fusion was identified in approximately 14.5% of a group of 62 patients with radiation-associated thyroid cancer who were exposed to radiation after a nuclear accident of Chernobyl [37].

### 4.3 Cancer of the central nervous system

#### 4.3.1 Neuroblastoma

Several reports found that NTRK receptors play an important role in the development of neuroblastomas. NTRK1/TrkA is expressed in tumors with favorable evolution, which have more chances of regression, or which respond favorably to usual therapy. High levels of *NTRK1* were correlated with favorable risk factors such as young age, histological type, etc. NTRK2/TrkB is expressed in tumors with unfavorable evolution, usually tumors that also have *MYCN* amplification. These tumors co-express a ligand for TrkB, BDNF, creating an autocrine pathway that causes drug resistance, angiogenesis, increased invasiveness, and a tendency to metastasize. Regarding TrkC, it was observed that the effects of its expression in medulloblastomas are similar to the effects of TrkA expression in neuroblastomas [10,11].

#### 4.3.2 Glioma

From a clinical point of view, it has been shown that gliomas that have fusions of the *NTRK* gene can affect any compartment of the central nervous system, being predominantly hemispherical in both the adult and pediatric populations. Also, gliomas with a low degree of differentiation and showing fusion of the *NTRK* gene may show an aggressive clinical course. It has been observed that the fusion of *NTRK* genes differs as a percentage depending on age. *NTRK2* is predominant in the pediatric population while *NTRK1* is found in the adult population [12].

### 4.4 Melanoma

*NTRK* fusions can also be seen in melanoma. In a study of 751 melanoma patients, 4 patients with metastatic malignant melanoma were identified in the lymph nodes, skin, colon and duodenum, and showed fusion of the *NTRK* gene [13]. The types of fusions associated with melanomas reported so far are *LMNA-NTRK1, TP53-NTRK1, ETV6-NTRK3, MYO5A-NTRK3 and MYH9-NTRK3* [13].

### 4.5 Hematological neoplasms

*NTRK* gene fusions are also found in hematologic neoplasms, such as the *ETV6-NTRK* fusion in acute myeloid leukemia, chronic eosinophilic leukemia, and Philadelphia-like acute chromosome lymphoblastic leukemia. Other fusions have also been found in patients with leukemia including two fusions of the *NTRK 2* gene (A203Tsi R458G) and two fusions of the *NTRK 3* gene (E176D and L449F). The therapeutic response of these neoplasms to the administration of TRK inhibitors such as larotrectinib and entrectinib is surprisingly favorable, and may provide a promise to this cancer population with a personalized treatment of all neoplasms with *NTRK* gene fusions [14].

## 5 NTRK fusions and emerging therapeutics

Targeting NTRK associated cancers has led to the development of two innovative therapies including larotrectinib and entrectinib which were approved by the Food and Drug Administration (FDA) in 2018 and 2019 respectively. Unlike entrectinib which is a multikinase inhibitor that targets TrkA, TrkB, TrkC, ROS1 and ALK1, larotrectinib is a selective pan-Trk inhibitor, being the first oral “tumor-agnostic” treatment and the first developed simultaneously for both adults and children [[15], [16], [17]]. Both treatments are administered orally, penetrate the blood-brain barrier and have demonstrated efficacy and safety in locally advanced, even metastatic, cancers that show *NTRK* gene fusions, regardless of location, type of fusion, histopathological type or age of the patient within the pediatric population [18]The identification of patients with neoplasms with *NTRK* gene fusions is constantly growing, especially with the advent of NTRK inhibitors, thus, allowing a personalized therapeutic approach. This has increased efficacy, good tolerance, acceptable route of administration with long-term favorable effects. Therefore, it is important to determine the fusion of *NTRK* genes in locally advanced or metastatic neoplasms, and beyond because of its potential as a predictive biomarker. By using this personalized, targeted, well-tolerated oncologic treatments such as entrectinib and larotrectinib multikinase inhibitors, improved outcomes may be achieved. This includes the stage of neoplasia that may decrease which can give the patient a chance for curative multimodal oncological and surgical treatment, even in stages that are initially advanced.

## 6 NTRK fusions as prognostic biomarkers

*NTRK* gene fusions are emerging biomarkers and their use in translational research and also for patients’ stratification is at its beginning. Particularly, *NTRK1-3* fusions with other genes were recently studied in several solid tumors harboring this signature as predictors of prognosis (Table 2) [[38], [39], [40], [41], [42], [43], [44], [45]]. In thyroid carcinomas, a cohort of 259 patients assessed *NTRK* chromosomal rearrangements and their correlation with histological patterns [39]. Indeed, patients with *NTRK* fusion-positive tumors had follicular growth feature, chronic thyroiditis with lymphocytic infiltration and lymphatic dissemination. In addition, carcinomas cases harboring *NTRK1* rearrangement were associated with multifocal and aggressive tumors. Remarkably, this genetic alteration was one of prognostic factors identified in this study in addition to tumor size, metastasis, and late *TP53* and *TERT* mutations [39]. In the pediatric population, these authors have also investigated these fusions in papillary thyroid carcinomas which may have distinct features including molecular profiles [43]. In this perspective, the authors investigated the genetic profile of these tumors in a cohort of 93 patients using DNA and RNA targeted next-generation sequencing. The most common identified altered genes were *RET* and *NTRK3*. In fact, fusions in *NTRK3* were found associated with follicular feature of this aggressive entity. On the other hand, molecular profiling *NTRK1/3* fusions in other pediatric tumors such as those of mesenchymal origin such as sarcomas is not well studied. Another recent study reviewed *NTRK* fusion positive sarcomas and showed, based on next-generation sequencing and fluorescence *in situ* hybridization, that they highly express Trk protein [41] which is responsible of the activation of a number of key downstream intracellular pathways, including the MAPK pathway (reviewed elsewhere: see: [46]). The authors also identified a fusion (*ETV6-NTRK3*) that is associated with good prognosis [41]. Thus, this suggests that not all *NTRK* fusions are predictive of aggressive behavior. Moreover, it seems that not all NTRK fusions are translating prognostic outcomes. In glioblastoma for example, Woo et al. demonstrated that *NTRK* fusions have no effects on prognosis [42]. However, other recent reports found that these alterations may be associated with acquired resistance to tyrosine kinase inhibitors and also with unfavorable survival outcomes, respectively, in non-small cell lung cancer and other multiple solid cancers [40,44]. Promisingly and in a recent advance, Rolfo et al. investigated a liquid biopsy-based approach to assess activating fusions of *NTRK* in circulating tumor DNA (ctDNA) from patients with advanced solid cancers [38]. These authors were able to identify NTRK1 fusions including some unique alterations that were correlated with those detected in corresponding tissues. Thus, highlighting the importance of ctDNA as a non-invasive screening method to select patients to benefit from targeted therapies [38].

**Table 2:** **Recent findings on the impact of NTRK fusions in cancer**.

Author/year	Study design	NTRK gene	Setting	Findings
Rolfo et al., 2022 [51]	Retrospective biomarker study	NTRK1	Multiple cancer types	-Liquid biopsy based on circulating tumor DNA (ctDNA) identified NTRK1 fusions including unique variants with high predictive value.
Pekova et al., 2021 [52]	Retrospective biomarker study	NTRK1 and NTRK3	Thyroid carcinoma	-NTRK fusion-positive patients had follicular growth pattern, chronic lymphocytic thyroiditis and lymph node metastases. -Carcinomas harboring NTRK1-rearrangements are more aggressive than NTRK3-positive tumors. -NTRK fusions are associated with survival outcomes.
Bazhenova et al., 2021 [53]	Retrospective biomarker study	NTRK1-3	Multiple cancer types	-Overall survival of patients with NTRK fusions might be reduced.
Kang et al., 2020 [54]	Retrospective biomarker study	NTRK1 and NTRK3	Pediatric sarcomas	-Childs with NTRK fusions highly expressed Trk protein. -Use of immunohistochemistry may help in diagnosing NTRK positive tumors. -Patients harboring ETV6-NTRK3 fusions fibrosarcoma had excellent prognosis.
Woo et al., 2020 [55]	Retrospective biomarker study	NTRK 2 and 3	Glioblastomas	-Glioblastomas can also harbor NTRK fusions but they do not affect prognosis.
Pekova et al., 2020 [56]	Cohort	NTRK3	Pediatric papillary thyroid carcinoma	-NTRK3 positive cases were associated with the follicular variant of this pediatric cancer.
Xia et al., 2020 [57]	Retrospective biomarker study	NTRK1	Non-small cell lung cancer	-NTRK1 positive fusion might be associated with acquired resistance to tyrosine kinase inhibitors.
Lasota et al., 2020 [58]	Case series	NTRK1 and NTRK3	Colon cancer	-Colorectal cancers with NTRK fusions are rare and distinct.

## 7 Conclusion

*NTRK* fusions in cancer are an emerging target in modern precision oncology. It is therefore important to determine the fusion of *NTRK* genes in locally advanced or metastatic neoplasms, and beyond. Ongoing clinical trials may provide better evidence to explore this target in the future.

## Ethical approval

Not needed for this mini-review.

## Sources of funding

None.

## Author contribution

CAM, AZ and DCB were responsible for writing of the manuscript. ICP and CGS were responsible for reviewing and editing of the manuscript. IDS, CGP, XB and VB made substantial contributions to the conception or design of the work. VTS were responsible for critically reviewing the manuscript. All Authors read and approved the final manuscript.

## Registration of research studies

Not needed for this narrative mini-review.

## Guarantor

Dr. Xenia Bacinschi (MD)

## Consent

No consents were needed.

## Declaration of competing interest

The authors state that they have no conflicts of interest for this case series.

## Provenance and peer review

Not commissioned, externally peer reviewed.

## Declaration of competing interest

The authors report no conflicts of interest.

## References

[1] Solomon J.P., Benayed R., Hechtman J.F., Ladanyi M. (2019 Nov 1). Identifying patients with NTRK fusion cancer. Ann. Oncol..

[2] Okamura R., Boichard A., Kato S., Sicklick J.K., Bazhenova L., Kurzrock R. (2018). Analysis of *NTRK* alterations in pan-cancer adult and pediatric malignancies: implications for NTRK-targeted therapeutics. JCO Precis Oncol.

[3] Li H., Yan S., Liu Y., Ma L., Liu X., Liu Y., Cheng Y. (2020 Sep). Analysis of NTRK mutation and clinicopathologic factors in lung cancer patients in northeast China. Int. J. Biol. Markers.

[4] Lassen U. (2019 Nov 25). How I treat *NTRK* gene fusion-positive cancers. ESMO Open.

[5] Amatu A., Sartore-Bianchi A., Siena S. (2016 Mar 18). *NTRK* gene fusions as novel targets of cancer therapy across multiple tumour types. ESMO Open.

[6] Doebele R.C., Drilon A., Paz-Ares L., Siena S., Shaw A.T., Farago A.F., Blakely C.M., Seto T., Cho B.C., Tosi D., Besse B., Chawla S.P., Bazhenova L., Krauss J.C., Chae Y.K., Barve M., Garrido-Laguna I., Liu S.V., Conkling P., John T., Fakih M., Sigal D., Loong H.H., Buchschacher G.L., Garrido P., Nieva J., Steuer C., Overbeck T.R., Bowles D.W., Fox E., Riehl T., Chow-Maneval E., Simmons B., Cui N., Johnson A., Eng S., Wilson T.R., Demetri G.D. (2020 Feb). Trial investigators. Entrectinib in patients with advanced or metastatic NTRK fusion-positive solid tumours: integrated analysis of three phase 1-2 trials. Lancet Oncol..

[7] Doebele R.C., Davis L.E., Vaishnavi A., Le A.T., Estrada-Bernal A., Keysar S., Jimeno A., Varella-Garcia M., Aisner D.L., Li Y., Stephens P.J., Morosini D., Tuch B.B., Fernandes M., Nanda N., Low J.A. (2015 Oct). An oncogenic NTRK fusion in a patient with soft-tissue sarcoma with response to the tropomyosin-related kinase inhibitor LOXO-101. Cancer Discov..

[8] Wong Victor, Dean Pavlick, Brennan Tim, Roman Yelensky, Crawford John, Ross Jeffrey S., Miller Vincent A., Malicki Denise, Stephens Philip J., Ali Siraj M. (January 2016). Hyunah ahn, evaluation of a congenital infantile fibrosarcoma by comprehensive genomic profiling reveals an *LMNA-NTRK1* gene fusion responsive to crizotinib. JNCI: J. Natl. Cancer Inst..

[9] Créancier L., Vandenberghe I., Gomes B., Dejean C., Blanchet J.C., Meilleroux J., Guimbaud R., Selves J., Kruczynski A. (2015 Aug 28). Chromosomal rearrangements involving the NTRK1 gene in colorectal carcinoma. Cancer Lett..

[10] Bongarzone I., Pierotti M.A., Monzini N., Mondellini P., Manenti G., Donghi R., Pilotti S., Grieco M., Santoro M., Fusco A. (1989 Dec). High frequency of activation of tyrosine kinase oncogenes in human papillary thyroid carcinoma. Oncogene.

[11] Farago Anna F., Le Long P., Zheng Zongli, Muzikansky Alona, Alexander Drilon, Patel Manish, Bauer Todd M., Liu Stephen V., Ou Sai-Hong I., Jackman David, Costa Daniel B., Multani Pratik S., Li Gary G., Hornby Zachary, Chow-Maneval Edna, Luo David, Lim Jonathan E., Iafrate Anthony J., Shaw Alice T. (2015). Durable clinical response to entrectinib in NTRK1-rearranged non-small cell lung cancer. J. Thorac. Oncol..

[12] Frattini V., Trifonov V., Chan J.M., Castano A., Lia M., Abate F., Keir S.T., Ji A.X., Zoppoli P., Niola F., Danussi C., Dolgalev I., Porrati P., Pellegatta S., Heguy A., Gupta G., Pisapia D.J., Canoll P., Bruce J.N., McLendon R.E., Yan H., Aldape K., Finocchiaro G., Mikkelsen T., Privé G.G., Bigner D.D., Lasorella A., Rabadan R., Iavarone A. (2013 Oct). The integrated landscape of driver genomic alterations in glioblastoma. Nat. Genet..

[13] Kim J., Lee Y., Cho H.J., Lee Y.E., An J., Cho G.H., Ko Y.H., Joo K.M., Nam D.H. (2014 Mar 19). NTRK1 fusion in glioblastoma multiforme. PLoS One.

[14] Siena S., Drillon A., Ou S.H. (2015). Entrectinib (RXDX-101), an oral pan-Trk, ROS1, and ALK inhibitor in patients with advanced solid tumors harboring gene rearrangements. Eur. J. Cancer.

[15] Shah N., Lankerovich M., Lee H. (2013). Exploration of the gene fusion landscape of glioblastoma using transcriptome sequencing and copy number data. BMC Genom..

[16] Wu G., Diaz A.K., Paugh B.S. (2014). The genomic landscape of diffuse intrinsic pontine glioma and pediatric non-brainstem high-grade glioma. Nat. Genet..

[17] Vaishnavi A., Capelletti M., Le A.T. (2013). Oncogenic and drug-sensitive NTRK1 rearrangements in lung cancer. Nat. Med..

[18] Ross J.S., Wang K., Gay L. (2014). New routes to targeted therapy of intrahepatic cholangiocarcinomas revealed by next-generation sequencing. Oncol..

[19] Greco A., Miranda C., Pierotti M.A. (2010). Rearrangements of NTRK1 gene in papillary thyroid carcinoma. Mol. Cell. Endocrinol..

[20] Wiesner T., He J., Yelensky R., Esteve-Puig R., Botton T., Yeh I., Lipson D., Otto G., Brennan K., Murali R., Garrido M., Miller V.A., Ross J.S., Berger M.F., Sparatta A., Palmedo G., Cerroni L., Busam K.J., Kutzner H., Cronin M.T., Stephens P.J., Bastian B.C. (2014). Kinase fusions are frequent in Spitz tumours and spitzoid melanomas. Nat. Commun..

[21] Braghiroli M.I. (2016). Genomic profiling and efficacy of anti-EGFR therapy in appendiceal adenocarcinoma. J. Clin. Oncol..

[22] Stransky N., Cerami E., Schalm S. (2014). The landscape of kinase fusions in cancer. Nat. Commun..

[23] Tognon C., Knezevich S.R., Huntsman D. (2002). Expression of the ETV6-NTRK3 gene fusion as a primary event in human secretory breast carcinoma. Cancer Cell.

[24] Makretsov N., He M., Hayes M., Chia S., Horsman D.E., Sorensen P.H., Huntsman D.G. (2004 Jun). A fluorescence in situ hybridization study of ETV6-NTRK3 fusion gene in secretory breast carcinoma. Genes Chromosomes Cancer.

[25] Lagree M., Toutain F., Revillon Y., Gaussin G., Marie-Cardine A., Lemoine F., Vannier J.P. (2011 Jan). Fibrosarcome infantile récidivant et métastatique: à propos d'un cas [Recurrent and metastatic infantile fibrosarcoma: a case report]. Arch. Pediatr..

[26] Knezevich S.R., McFadden D.E., Tao W. (1998). A novel ETV6-NTRK3 gene fusion in congenital fibrosarcoma. Nat. Genet..

[27] Morerio C., Rapella A., Rosanda C., Lanino E., Lo Nigro L., Di Cataldo A., Maserati E., Pasquali F., Panarello C. (2004 Jul 15). MLL-MLLT10 fusion in acute monoblastic leukemia: variant complex rearrangements and 11q proximal breakpoint heterogeneity. Cancer Genet. Cytogenet..

[28] Leeman-Neill R.J., Kelly L.M., Liu P., Brenner A.V., Little M.P., Bogdanova T.I., Evdokimova V.N., Hatch M., Zurnadzy L.Y., Nikiforova M.N., Yue N.J., Zhang M., Mabuchi K., Tronko M.D., Nikiforov Y.E. (2014 Mar 15). ETV6-NTRK3 is a common chromosomal rearrangement in radiation-associated thyroid cancer. Cancer.

[29] Eguchi Mariko, Eguchi-Ishimae Minenori, Tojo Arinobu, Morishita Kazuhiro, Suzuki Katsuyuki, Sato Yuko, Kudoh Shiori, Tanaka Kimio, Setoyama Misao, Nagamura Fumitaka, Asano Shigetaka, Kamada Nanao (1999). Fusion of ETV6 to neurotrophin-3 receptor TRKC in acute myeloid leukemia with t(12;15)(p13;q25). Blood.

[30] Knezevich S.R., McFadden D.E., Tao W., Lim J.F., Sorensen P.H. (1998 Feb). A novel ETV6-NTRK3 gene fusion in congenital fibrosarcoma. Nat. Genet..

[31] Brenca M., Rossi S., Polano M., Gasparotto D., Zanatta L., Racanelli D., Valori L., Lamon S., Dei Tos A.P., Maestro R. (2016 Mar). Transcriptome sequencing identifies ETV6-NTRK3 as a gene fusion involved in GIST. J. Pathol..

[32] Skálová A., Weinreb I., Hyrcza M., Simpson R.H., Laco J., Agaimy A., Vazmitel M., Majewska H., Vanecek T., Talarčik P., Manajlovic S., Losito S.N., Šteiner P., Klimkova A., Michal M. (2015 Mar). Clear cell myoepithelial carcinoma of salivary glands showing EWSR1 rearrangement: molecular analysis of 94 salivary gland carcinomas with prominent clear cell component. Am. J. Surg. Pathol..

[33] Hechtman J.F., DeMatteo R., Nafa K., Chi P., Arcila M.E., Dogan S., Oultache A., Chen W., Hameed M. (2015 Aug). Additional primary malignancies in patients with gastrointestinal stromal tumor (gist): a clinicopathologic study of 260 patients with molecular analysis and review of the literature. Ann. Surg Oncol..

[34] Cocco E., Scaltriti M., Drilon A. (2018 Dec). NTRK fusion-positive cancers and TRK inhibitor therapy. Nat. Rev. Clin. Oncol..

[35] Botnariuc I., Ilie S.M., Trifanescu O.G., Bacinschi X.E., Curea F., Anghel R.M. (2017 Apr-Jun). Predictive circulating markers for anthracycline chemotherapy in non-metastatic breast cancer. Acta Endocrinol..

[36] Ricciuti B., Genova C., Crinò L., Libra M., Leonardi G.C. (2019 Apr 30). Antitumor activity of larotrectinib in tumors harboring *NTRK* gene fusions: a short review on the current evidence. OncoTargets Ther..

[37] Wiesner T., He J., Yelensky R., Esteve-Puig R., Botton T., Yeh I., Lipson D., Otto G., Brennan K., Murali R., Garrido M., Miller V.A., Ross J.S., Berger M.F., Sparatta A., Palmedo G., Cerroni L., Busam K.J., Kutzner H., Cronin M.T., Stephens P.J., Bastian B.C. (2014). Kinase fusions are frequent in Spitz tumours and spitzoid melanomas. Nat. Commun..

[38] Rolfo C., Drilon A., Hong D. (2022 Feb). NTRK1 Fusions identified by non-invasive plasma next-generation sequencing (NGS) across 9 cancer types. Br. J. Cancer.

[39] Pekova B., Sykorova V., Mastnikova K. (2021 Apr 16). NTRK fusion genes in thyroid carcinomas: clinicopathological characteristics and their impacts on prognosis. Cancers.

[40] Bazhenova L., Lokker A., Snider J., Castellanos E., Fisher V., Fellous M., Nanda S., Zong J., Keating K., Jiao X. (2021 May). TRK fusion cancer: patient characteristics and survival analysis in the real-world setting. Targeted Oncol..

[41] Kang J., Park J.W., Won J.K., Bae J.M., Koh J., Yim J., Yun H., Kim S.K., Choi J.Y., Kang H.J., Kim W.S., Shin J.H., Park S.H. (2020 Sep 21). Clinicopathological findings of pediatric NTRK fusion mesenchymal tumors. Diagn. Pathol..

[42] Woo H.Y., Na K., Yoo J., Chang J.H., Park Y.N., Shim H.S., Kim S.H. (2020 Oct). Glioblastomas harboring gene fusions detected by next-generation sequencing. Brain Tumor Pathol..

[43] Pekova B., Sykorova V., Dvorakova S., Vaclavikova E., Moravcova J., Katra R., Astl J., Vlcek P., Kodetova D., Vcelak J., Bendlova B. (2020 Dec). RET, NTRK, ALK, BRAF, and MET fusions in a large cohort of pediatric papillary thyroid carcinomas. Thyroid.

[44] Xia H., Xue X., Ding H., Ou Q., Wu X., Nagasaka M., Shao Y.W., Hu X., Ou S.I. (2020 May). Evidence of NTRK1 fusion as resistance mechanism to EGFR TKI in EGFR+ NSCLC: results from a large-scale survey of NTRK1 fusions in Chinese patients with lung cancer. Clin. Lung Cancer.

[45] Lasota J., Chłopek M., Lamoureux J. (2020 Feb). Colonic adenocarcinomas harboring NTRK fusion genes: a clinicopathologic and molecular genetic study of 16 cases and review of the literature. Am. J. Surg. Pathol..

[46] Weiss L.M., Funari V.A. (2021 Jun). NTRK fusions and Trk proteins: what are they and how to test for them. Hum. Pathol..

